# Development of Gaze Following Abilities in Wolves (*Canis Lupus*)

**DOI:** 10.1371/journal.pone.0016888

**Published:** 2011-02-23

**Authors:** Friederike Range, Zsófia Virányi

**Affiliations:** 1 Department of Cognitive Biology, University of Vienna, Vienna, Austria; 2 Wolf Science Center, Ernstbrunn, Austria; University of Alberta, Canada

## Abstract

The ability to coordinate with others' head and eye orientation to look in the same direction is considered a key step towards an understanding of others mental states like attention and intention. Here, we investigated the ontogeny and habituation patterns of gaze following into distant space and behind barriers in nine hand-raised wolves. We found that these wolves could use conspecific as well as human gaze cues even in the barrier task, which is thought to be more cognitively advanced than gazing into distant space. Moreover, while gaze following into distant space was already present at the age of 14 weeks and subjects did not habituate to repeated cues, gazing around a barrier developed considerably later and animals quickly habituated, supporting the hypothesis that different cognitive mechanisms may underlie the two gaze following modalities. More importantly, this study demonstrated that following another individuals' gaze around a barrier is not restricted to primates and corvids but is also present in canines, with remarkable between-group similarities in the ontogeny of this behaviour. This sheds new light on the evolutionary origins of and selective pressures on gaze following abilities as well as on the sensitivity of domestic dogs towards human communicative cues.

## Introduction

One central feature of social life and communication in humans is the monitoring of others' head and eye orientation (gaze) [1]. The abilities to coordinate with others and look in the same direction (gaze following) or at a specific target (joint attention) develop early during ontogeny [2], and are considered to be a key step towards an understanding of mental states like attention and intention [3], [4]. Due to these theoretical implications and in order to understand the evolutionary roots of such capabilities, gaze-following abilities in non-human animal species have recently received increased attention [5].

Interestingly, while most animals have difficulties in interpreting the gaze of others as a communicative intentional cue and fail to choose a food container indicated by the gaze of a cooperative partner [6], animals are quite successful in using others' gaze to detect significant events in their environment. These later gaze following abilities have been observed in several animal species including apes [7], [8] and monkeys [9]–[11], but also other mammalian species such as ungulates [12], bird species [13], [14] and a red-footed tortoise (*Geochelone carbonaria*) [15].

However, whether or not subjects follow the gaze of others to detect environmental effects depends on the paradigm used to test gaze-following abilities. While several species have been shown to successfully follow another's gaze into distant space (e.g. ravens, *Corvus corax* [13], apes [7], domestic goats, *Capra hircus* [12], Northern bald ibises, *Geronticus eremita* [16]), only great apes [7] and two corvid species (ravens [13] and rooks, *Corvus frugilegus* [14]) have been reported to successfully follow another's gaze geometrically around a visual barrier e.g. by repositioning themselves to follow a gaze cue when faced with a barrier blocking their view. Two recent studies, for example, reported negative results for geometrical gaze following in the bald ibis [16] and the common marmoset, *Callithrix jacchus* [17].

This mosaic of results is strongly connected to theoretical considerations concerning the underlying cognitive mechanism of gaze following. Povinelli and Eddy [18] suggest that gaze following into distant space may be a socially facilitated orientation response (i.e. a predisposition to look where others are looking). This would probably require no more than an intrinsic tendency to co-orient with others, combined with associative learning [19], [20]. However, when faced with a barrier blocking their view, individuals need to reposition themselves to look behind the obstacle thus assessing the difference in visual perception between the cue-giver and themselves. This may either be achieved by mentally representing the looker's visual perspective [18] or by learning how visual barriers impair perceptions [19]. It has been hypothesized that especially species with high levels of cooperative and competitive interactions may develop the ability to track another's gaze around obstacles [21], whereas the capability to follow another's gaze into distant space is likely to be adaptive for most socially living vertebrates since it will allow detecting predators or food resources earlier e.g. [22], [23].

Results on the development of gaze following abilities as well on habituating to others' gaze cues seem to have confirmed that the two different modes of gaze following (into distant space and behind barriers) may reflect different cognitive mechanisms [18], [19]. First, developmental data showed that ravens responded to others' gazing into distant space soon after fledging but could track the experimenter's gaze behind a visual barrier only 4 months later [21]. This suggests that following another's gaze into distant space may be a predisposition to respond to the visual behavior of others, which after being shaped by learning may allow for gaze following around the barrier later in development (‘low-level model’ [18]; ‘orienting-response model’ [24]). Second, the authors found that ravens also stopped responding to gaze cues that did not target anything of interest. The same rapid habituation has also been found in several primate species at least when testing adult animals [25]; but see [10]. Interestingly, while the ravens quickly ceased responding to the model's repeated gaze cues into distant space, they did not habituate to repeated gaze cues directed behind a barrier. This differential habituation pattern of the two modes of gaze following suggests again that they rely on different cognitive mechanisms. Beyond habituation, a flexible deployment of gaze following is demonstrated by animals when readily following the gaze of not only conspecifics but also of human partners. Primates as well as ravens that grew up close to humans and regularly interacted with their handlers have been found to follow human gaze, though in ravens this ability developed later than following the gaze of conspecifics [7], [21]. To date, nothing is known about the development, the generalizability to humans and the habituation pattern of gaze following behind barriers in any mammalian species, which would be needed to better understand the cognitive mechanisms underlying the two modes of gaze following.

Wolves (*Canis lupus*), the ancestors of domestic dogs, are well known for their cooperative hunting [26]. Visual coordination, including following their partner's gaze into distant space and around barriers, should thus be very adaptive to their survival. It has been hypothesized, however, that, compared to domestic dogs, wolves may be less ready to accept humans as social partners [27], [28]. In line with this argument, one can expect that, similarly to ravens, wolves follow the gaze of humans later in development than the gaze of conspecifics. Therefore, in experiment 1, we tested the wolves across several ages to determine if they follow the gaze of both a human experimenter and a conspecific partner around a barrier, and, if so, when this ability emerges during development. Both to verify that wolves have the ability to follow humans' gaze direction as shown in experiment 1 and to determine the possible mechanisms involved, experiment 2 was designed to test the wolves' ability to follow the gaze of a human experimenter into distant space. According to the theoretical considerations outlined above, we expected this ability to develop earlier during ontogeny than following human gaze around the barrier. To gather additional information in regard to the underlying mechanisms, we also compared the habituation pattern of the two gaze following modalities using a human experimenter. Based on the only available data [21], we expected that wolves would quickly cease responding to repeated gaze cues into distant space but would show no habituation to repeated trials in the barrier task.

## General Methods

No special permission for use of animals (wolves) in such socio- cognitive studies is required in Austria. The relevant committees that allow to run research without special permissions regarding animals are: Tierversuchskommission am Bundesministerium für Wissenschaft und Forschung (Austria). The person shown in the photo gave written consent to the publication of the photo.

### (a) Subjects

All wolves (n = 9) that participated in this study originated from North America and were born in captivity. Three wolves (2 males, 1 female) from two different litters were born at Herberstein Zoo, Styria, Austria in May 2008. Six additional wolves from four different litters were raised in May 2009. Two brothers were obtained from the Basel Zoo, Switzerland; the other four animals (one brother-sister pair, 1 unrelated male, 1 unrelated female) were born at the Triple D Farm, Montana, USA. All of them were hand-raised in peer groups at the Wolf Science Center (www.wolfscience.at) after being separated from their mothers in the first 10 days after birth. They were bottle-fed and later hand-fed by humans and had continuous access to humans the first 5 months of their life. When the second generation was five months old, they were introduced to the pack of the 1.5 year-old wolves.

From this age on, there were no humans continuously present in the enclosure, but the wolves participated in training and/or cognitive and behavioural experiments at least once a day and hence had intensive social contact with humans. Also, five adult dogs of various breeds were present during the hand-raising of the wolves. They established close relationships with the wolves and until the end of this study all wolves readily submitted to the dogs.

During puppyhood, the animals were kept in a 1000 m^2^ outside enclosure with access to an indoor room (puppy room), where the hand-raisers, one at a time spent the nights with them. At five months they were moved to a 3000 m^2^ enclosure. The enclosures were equipped with trees, bushes, logs and shelters. Water for drinking was permanently available. The wolves received a diet of meat, fruits, milk products and dry food throughout the study period. During the first months of their lives, they were fed several times per day, which was slowly reduced to being fed major meals twice or three times per week according to their natural rhythm. All animals received intensive obedience training like sit, down, roll-over and eye contact on a daily basis using the clicker (operant conditioning with a secondary reinforcer). This training assures that the wolves are cooperative and attentive towards humans and also allows veterinary checks without sedating the animals. In the eye contact training, the wolves were rewarded for looking into the trainer's eyes. The trainer was looking only at the animals, and even if she occasionally got distracted during the training and looked to another direction, the wolves were never rewarded for following the trainer's gaze. All animals participated in various behavioural tests every week, where they were also rewarded with food. A testing room (6×10×6 m) next to the enclosures allowed for training and testing the animals in isolation from the pack. All wolves were worked in separation from the other wolves on a daily basis. Participation in all training and testing sessions was voluntary and all wolves competed with each other to have access to the testing room to interact with the experimenters.

### (b) General procedure

In general, we tested subjects for their abilities to follow looks of a human and a dog demonstrator behind a barrier once per month starting when subject were 16 weeks old (experiment1a). Shortly before experiment 1 at the age of 14 weeks, we started to test wolves' abilities to follow human gaze into distant space (experiment 2a). These tests were repeated every three weeks. In both treatments, a habituation study followed after acquisition of both abilities (experiments 1b, 2b). Tests were conducted individually in the indoor puppy room, in the indoor testing room or later on in the puppy enclosure (after the animals had moved to the pack enclosure). In Experiments 1a and 2a testing location was varied from test to test to avoid habituation to the task.

Studies were conducted by one of three hand-raisers with a second familiar experimenter assisting for videotaping during experiments or holding the wolf (in the barrier task). The experimenter, the wolf and the experimental set-up were visible on each video recording. For the dog demonstration in the barrier task, three (two females, one male) of the 5 dogs, who participated in raising the wolves were used as demonstrators. In experiments 1a and b, a wooden board (90° angle to the stone wall of the testing room or puppy enclosure) or standard enclosure equipment such as big rocks functioned as a visual barrier for the subjects. All gaze cues (human and dog) involved turning the head and the eyes to the side while the body stayed in the same position oriented toward the subject.

## Experiment 1: Gaze following around the barrier

In experiment 1, we aimed at testing if wolves would follow the gaze of a human and/or a conspecific experimenter around a barrier and how this ability develops over ontogeny. Furthermore, we tested if the animals habituated to repeated trials.

### Methods

#### Experiment 1a - Ontogeny

The experiment started when the animals were 16 weeks old. The human and dog demonstration trials were each carried out 3 times in two consecutive months. At that age, all subjects had reached full mobility and had been successfully trained on establishing eye contact upon hearing the command “look” (see Subjects and Methods of experiment 2a)**.** Each session consisted of one test and one control trial per wolf, carried out in a random order. At each age two sessions were conducted, one with a dog and one with a human demonstrator with a 2 to 4 day break between. The sequence of dog and human demonstrator was counterbalanced between and within wolves. Wolves were tested individually and had the possibility to inspect the whole test area before the onset of the experiment. At 5 months of age, only 7 of the 9 animals could be tested due to unwillingness of the animals to participate in the study (see discussion).

**Figure 1:**
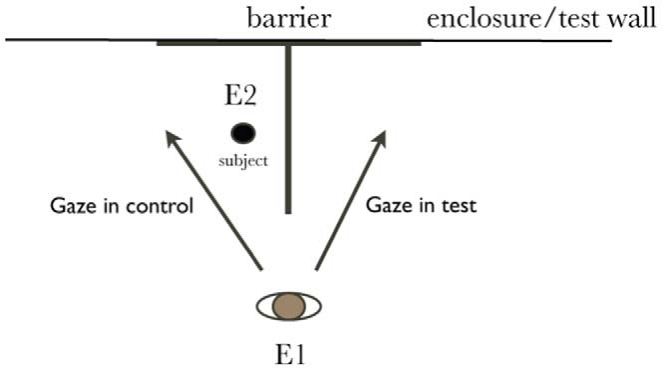
Layout of the barrier test, showing the position of the human E 1 and the start position of the test subject. E2 held the subject on the collar or leash until the gaze cue was given. The arrows indicate where E 1 looked in the test and control conditions respectively.

Human demonstration trials: E 1 was positioned in line with the barrier (Figure 1). E2 had the wolf on the collar and stood on one side of the barrier so that the subject's view to the other side of the barrier was blocked. E1 gave the command for eye contact ‘look’; as soon as the wolf established eye contact, E1 gave the gaze cue for 15 seconds. In control trials, E gazed at the subject's side of the barrier, without looking directly at the animal. In the test trials, E gazed at the other side of the barrier e.g. the wolf could not see what the experimenter was looking at on the other side of the barrier and would have to walk around the barrier to follow the gaze of the experimenter. As soon as the cue was given, E2 let go off the collar. No warm-up trials were conducted and no reward was present on the other side of the barrier during the human demonstration trials.

Dog demonstration trials: The dog was positioned in line with the barrier as E1 in the human demonstration trials. E2 had the subject on the collar or leash and stood on one side of the barrier so that the subject's view to the other side of the barrier was blocked. E1 was standing behind E2 and the wolf and commanded the dog to look at her (control trials) or to look on the other side of the barrier (test trials) when the wolf was looking at the dog. If the wolf was still looking towards the dog, when the command was carried out, the wolf was released. If not, we repeated the procedure until the wolf was looking towards the dog when the dog indicated the other side. In order to get the dog to really focus on the other side of the barrier (a trained head movement might have a very different quality than if the dog really knows that something is there), we put out a piece of dry food on the other side of the barrier, which was shown to the dog before each test trial while a second experimenter distracted the wolf. Thus, the command used to get the dog to focus on the food was ‘look at the food’. In contrast to the human demonstration, however, the dog looked at the other side of the barrier for shorter time periods before looking back at E2, but repeated its head turn several times during the 15 seconds. In control trials, the same type of food reward was also placed on the other side of the barrier while the wolf was distracted, but we did not show it to the dog explicitly. In the control trials the trained command was ‘look at me’. Following this command was rather easy for the dogs and they made only few mistakes. In these few cases we stopped the trial and repeated it later. Please see Video S1 for a demonstration.

The sequence of test and control trials, the side of the barrier on which the subject was positioned and whether an animal was tested first with the dog or human demonstrator was randomized across all sessions. Gaze following was considered if the subject moved around the barrier and oriented to the 1.2×1.2 m area on the indicated side of the barrier within 15 s (15 s presentation); subjects could change sides by walking around the barrier.

#### Experiment 1b - Habituation

Six wolves were tested 2 months after their last gaze following around the barrier experiments (after experiment 1a was finished), while the other three were tested 14 months later. For all of them it meant a week delay after the habituation experiment of gaze following into distant space (experiment 2b). Again, the test procedure was similar to that in experiment 1a with the exception that now several test cues were given in a row instead of one test and one control trial. In the very first trial a piece of dry food was positioned on the other side of the barrier to get the animal motivated to participate in further trials. The time interval between cues was set at 15 s or until the wolf was again in the correct position. We continued giving the cues until a subject had not responded in 3 consecutive trials but at least 7 times. The habituation was only carried out with a human demonstrator.

#### Analysis

Videos were coded using the Solomon coder software (Solomon Coder beta 10.05.06). Gaze following was coded based on the wolves' distinct orientation behavior: walking around the barrier and looking on the other side. We used two different measurements to describe the behavior of the animals. First, we analyzed the *immediate* response i.e. whether or not (1/0) the animals walked around the barrier to look on the other side within 5 seconds of the cue demonstration. We used 5 seconds to define the immediate response and not 2 seconds as has been done in previous studies analyzing gaze patterns [29], [30], since the animals had to move around the barrier first. Moreover, as a novel measurement, we analyzed the latency to visit the other side of the barrier over the entire test duration, since several wolves often first walked up to the experimenter giving the cue and greeted her. Thus, we feel that the latency to walk around the barrier is also an important measurement describing the response of the animals. Maximum latency was set at 15 s. The time resolution was 0.10 seconds. Twenty per cent of the trials were scored independently by a second observer to assess inter-observer reliability (Cohen Kappas: look around: 0.95). To investigate the performance of the wolves, we used McNemar tests to compare single test and control trials. Because of the 1/0 sampling method, we used Cochran's Q test for comparisons of more than two habituation trials [31]. To analyze latencies, we used non-parametric statistics since the data were not normally distributed based on the Kolmogorov–Smirnov test. Statistical analyses were performed in Instat 3. Results are given for two-tailed tests and alpha was set at 0.05. Trends are reported for 0.1>p>0.05.

### Results

#### 1a - Ontogeny

**Figure 2:**
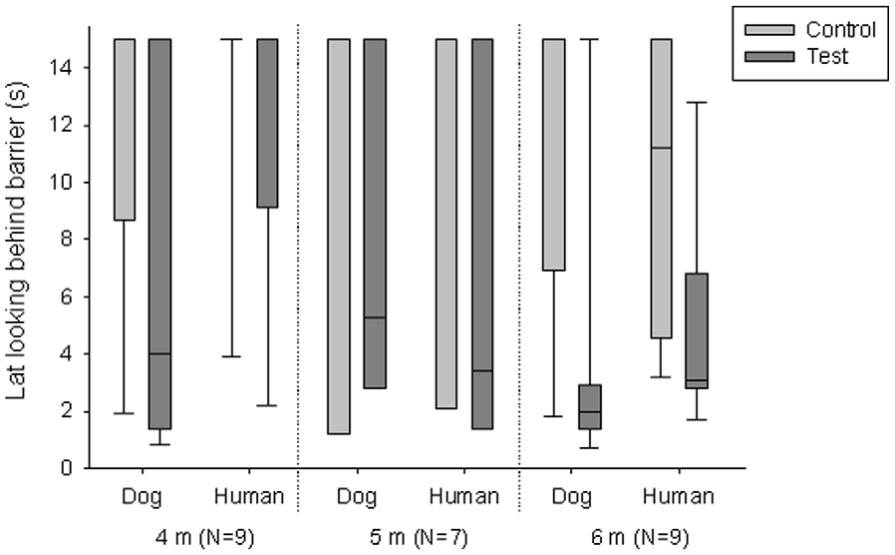
Box plots showing the latency of wolves to look behind the barrier in the control and test trials according to whether a dog or a human demonstrated the gaze cue. Each test was repeated at the age of 4, 5 and 6 months of age. Shaded boxes represent the interquartile range, bars within shaded boxes are median values and whiskers indicate the 5th and 95th percentile.

We found that the wolves walked around the barrier and looked on the other side significantly more often in the test trials than in the control trials only at the age of 6 months of age during the dog demonstration (McNemar test: N = 9, p<0.05) and tended to do so in the human demonstration condition (McNemar test: N = 9, p<0.1) (Table 1). At earlier ages no significant differences were found between the two conditions (all p>0.1). Analyzing latencies we found similar results, namely that at the age of 4 months, the subjects showed no significant difference in their latency to look on the other side of the barrier in the test trials compared to the control trials neither with the dog nor human demonstrator (Wilcoxon matched-pairs signed ranks test: human demonstrator: N = 9 (6 ties), T = 1, p = 0.500; dog demonstrator: N = 9 (3 ties), T = 4, p = 0.219, Figure 2). Interestingly, while in the human demonstrator condition only 3 animals ever looked on the other side of the barrier, six did so in the dog demonstration. No significant difference could be found between the test and the control trials at the age of 5 months (Wilcoxon matched-pairs signed ranks test: human demonstrator: N = 7 (1 tie), T = 5, p = 0.3125; dog demonstrator: N = 7 (2 ties), T = 7, p = 0.99, Figure 2). With 6 months of age, however, all animals participated again in the study and we found a significant difference in the latency to look on the other side of the barrier between the test and control trial irrespective of the demonstrator (Wilcoxon matched-pairs signed ranks test: human demonstrator: N = 9, T = 1.5, p = 0.008; dog demonstrator: N = 9 (1 tie), T = 2, p = 0.023, Figure 2).

**Table 1:** Individual performance of the 9 wolves in the gaze following around barrier task in relation to both analyzed variables.

	Dog Demonstrator						Human Demonstrator					
	4 months		5 months		6 months		4 months		5 months		6 months	
Ind	1/0	Lat	1/0	Lat	1/0	Lat	1/0	Lat	1/0	Lat	1/0	Lat
Ar	+	+	NA	NA	+	+	-	-	NA	NA	+	+
Ka	-	+	-	-	-	-	-	-	NA	NA	-	+
Sh	+	+	NA	NA	+	+	+	+	+	+	+	+
Ta	-	-	-	-	-	-	-	-	-	-	+	+
Na	-	-	-	-	+	+	-	-	-	-	-	-
Ge	-	-	+	+	+	+	-	-	+	+	-	+
Yu	-	-	-	-	+	+	-	-	-	+	-	+
Ap	+	+	-	-	-	+	-	-	-	+	+	+
Ch	+	+	-	+	+	+	+	+	+	+	-	+

1/0:+ =  following gaze in test but not control trial.

- = following the gaze in both trials *or* in none ( = tie).

Lat:+ =  shorter latency in test than control.

- = longer latency in test than control *or* no reaction in either trial ( = tie).

NA =  not available.

1/0 refers to whether or not the subject checked the other side of the barrier within the first 5 seconds of the cue presentation. Latency (Lat) refers to if the subject followed the gaze cue earlier in the test trial than the control trial.

#### 1b - Habituation

**Figure 3:**
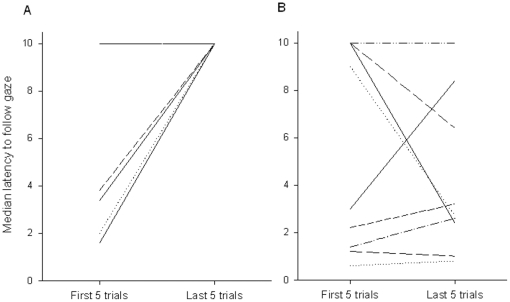
Median latency of individual wolves to follow the gaze in the first 5 and last 5 trials of the habituation experiments. A: Gaze following around the barrier; B: Gaze following into distant space.

When comparing the latency of the gaze following in the first trial of the habituation experiment with the latency of the last test trial of the barrier test at the age of 6 months, no significant difference was found (Wilcoxon matched-pairs signed ranks test: N = 9, T = 9, p = 0.129). However, compared to the last control trial at the age of 6 months, the animals checked the other side significantly earlier in the first habituation trial (Wilcoxon matched-pairs signed ranks test: N = 9, T = 0, p = 0.004). Overall, we found a significant increase in the wolves' latency to look on the other side of the barrier from the 5 first trials to the last 5 trials (Wilcoxon matched-pairs signed ranks test: N = 9, T = 0, p = 0.004) (Figure 3a). Four of the nine animals checked the other side of the barrier only a single time before stopping to respond to the cue either by leaving the area completely, staying on their side of the barrier, interacting with the experimenter or laying down to sleep. Similar results we found when analyzing the immediate response e.g. a steady and significant decline in the occurrence of response in the barrier test within the first 5 seconds after the gaze cue was given (Cochran's Q: Q_4_ = 38.44, N = 9, p>0.001).

### Discussion

The results of experiment 1 show that gaze following around the barrier appears in wolves reliably only at the age of 6 months. While the latency to look on the other side of the barrier was significantly shorter in both the human and the dog demonstration test trials compared to the control trials, we only found a significant difference in the dog demonstration trials when analyzing the response within the first 5 seconds. There could be two different explanations for the different results of the two analyses: First, some animals often went to the human experimenter first for greeting before actually looking on the other side of the barrier, which postponed the measured response behaviour. Alternatively, the response of the wolves may be somewhat stronger to conspecific gaze than to human gaze. This possible explanation is supported by the fact that 5 of the 9 wolves responded already at the age of 4 months to the gaze cue of the dog demonstrator, moving around the barrier either only in the test trial or in the test trial earlier than in the control trial. In comparison only two animals checked the other side of the barrier preferably in the test compared to the control trial after the human demonstration. This suggests that the ability to follow the gaze of a conspecific around a barrier might already be present at an earlier age at least in some animals. Unfortunately, around the age of 5 months, wolves seem to undergo a period of uncooperativeness at least towards humans. It was impossible to get 2 of the 9 animals to look at the demonstrator for more than a second at this age and consequently, these animals could not be tested. At the age of 6 months, the animals were much more cooperative again, and 7 of 9 animals responded to the dog's and 8 of 9 animals to the human's gaze in the test trials but later or not at all in the control trials. Overall the results indicate that by 6 months of age, wolves reliably follow the gaze of conspecifics and to a certain degree also of humans around a barrier.

Although training the wolves to accept eye contact enabled us to conduct the tests, such training does not appear to explain the results obtained. The eye-contact training was never conducted with a conspecific partner and also with a human partner the wolves were only rewarded for looking into an experimenter's eyes during the training. Since they never received reward for following a gaze cue, we most likely trained them *against* following the gaze of the experimenter rather inhibiting than promoting their spontaneous response. This may also explain the slightly weaker response to human gaze compared to conspecific gaze.

Accordingly it seems that at the age of 6 months, wolves take the physical, view-blocking feature of barriers into account. Whether this ‘understanding’ requires an attribution of mental states to the demonstrator or whether this ability may develop through individuals' daily experiences with other that move in front of or behind obstacles is an open question [19].

Another result in support of the notion that the gaze following response in the barrier task was strongly influenced by learning is the rapid habituation of the wolves. In the barrier task, all wolves habituated within 10 trials, and 4 ceased responding already after the very first trial even though they did find a piece of food in that very first trial. This is in strong contrast to ravens that did not habituate to a repeatedly given cue within 5 trials in the barrier task [21]. Since in our barrier task, the wolves were free to investigate the other side of the barrier in each trial, the animals could quickly learn that the cue had no meaning and thus may have habituated rather quickly to the repeated cue. However, the fact that four animals ceased responding already after the very first trial when they found food suggest an even more elaborate understanding of the entire test situation, namely that after having checked the other side thoroughly in the first trial and having seen nobody approach this area, no further food could have been available in the subsequent trials. Thus, whether animals habituate to a certain gaze cue or not might be dependent of the cue used as well as on the actual test situation, and thus seems to be flexible.

## Experiment 2: Gaze following into distant space

In this experiment we aimed at testing whether wolves' ability to follow human into distant space develops earlier than following human gaze around a barrier and whether wolves would habituate to these gaze cues differently.

### Methods

#### Experiment 2a - Ontogeny

**Figure 4:**
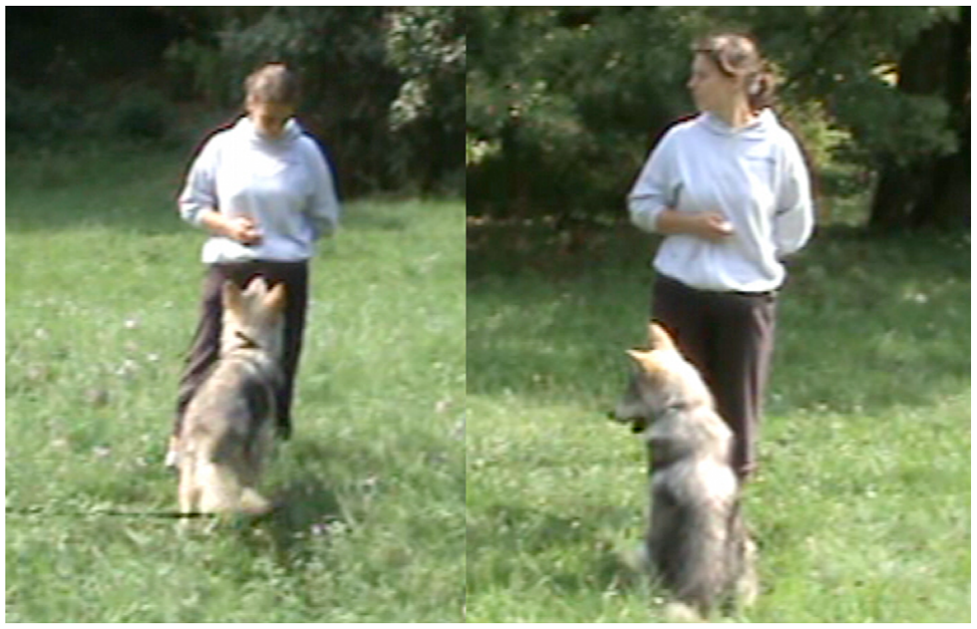
Gaze following into distant space. A: control trial; B: test trial.

Experiment 2a was started at the age of 14 weeks; these tests were repeated every three weeks for 3 months. Similarly to experiment 1a, a session consisted of one test and one control trial. At the start of each trial (test as well as control), the experimenter (E) established eye contact with the wolf by giving the trained command ‘look’ and rewarding the animal when it looked into her eyes. After 2 to 4 such warm-up trials, the experimenter asked once again for establishing eye contact. As soon as the subject looked into the eyes of the E, E gave a gaze cue. In the test trials, E looked to a point at a 90° angle (Figure 4). In control trials E looked to a point just next to the wolf instead of looking to the side (Figure 4). We decided not to look directly at the wolf in control trials, since this extended staring might have been perceived as a threat by the wolf. Neither the test nor the control trials were rewarded in any way. The order of control and test trials as well as the direction of the look cue was randomized. A look cue was given for ten seconds and the subject's response within the ten seconds (i.e., whether or not it looked) was noted later from the video records. The time interval between trials was at least 10 s; the exact time was dependent on the wolf's attention towards E. The video was recorded by the second experimenter standing 3–5 meters behind the wolf. Please see Video S2 for a demonstration.

#### Experiment 2b - Habituation

All wolves were tested 1 week before the habituation trials of experiment 1b. For six wolves this meant a 2-month delay after the last gaze following experiment (after experiment 1a was finished) and a 14 months delay for the other three wolves. The test procedure was similar to that in experiment 1a with the exception that ten look-aside (i.e. test) cues were given in a row instead of one test and one control trial. The time interval between cues was set at 15 s or until the wolf was again in the correct position. Before each cue eye contact was reinforced once or twice with a food reward to keep up the motivation of the animals to participate in the study.

#### Analysis

All trials were scored from videotapes using the Solomon coder software (Solomon Coder beta 10.05.06). We used head orientation to determine looking to the side. Since wolves do not have many predators coming from above and do not need to check for or communicate with conspecifics that are above them in the air or on trees such as birds or primates, we used the ‘look to the side’ cue that is more relevant for the wolves than a ‘look-up’ cue. However, the frequency of spontaneous look-ups is most likely much lower than looks to the side and thus the probability that subjects look somewhere during the 10 s of the cue, especially if the experimenter is just standing still and not engaging in any interactions, is much higher. On the other hand, subjects sometimes got distracted, looked somewhere else than the experimenter (to the ground, behind the experimenter etc), but at one point gazed back into the face of the experimenter and then followed the gaze cue. Accordingly we used again two different measurements to describe the reaction of the animals. First, in each test and control trial, we analyzed the immediate reaction of the wolf to the gaze cues by recording whether or not (1/0) the *first* detectable head turn of the subject followed the direction of the demonstrated head movement. An immediate response was defined as a response within 2 seconds after E looked to the side according to previous studies analyzing gaze patterns [29], [30]. Moreover, as a second measurement, we again analyzed the latency of looking into the direction of the demonstrated head movement of the test trial both in the test and in the control trial during the 10 seconds after the cue was given. Maximum latency was set at 10 s. The time resolution was 0.10 seconds. Twenty per cent of the trials were scored independently by a second observer to assess inter-observer reliability (Cohen Kappas: look-aside: 0.82). The same statistical tests were used as in experiment 1.

### Results

#### 1a - Ontogeny

**Figure 5:**
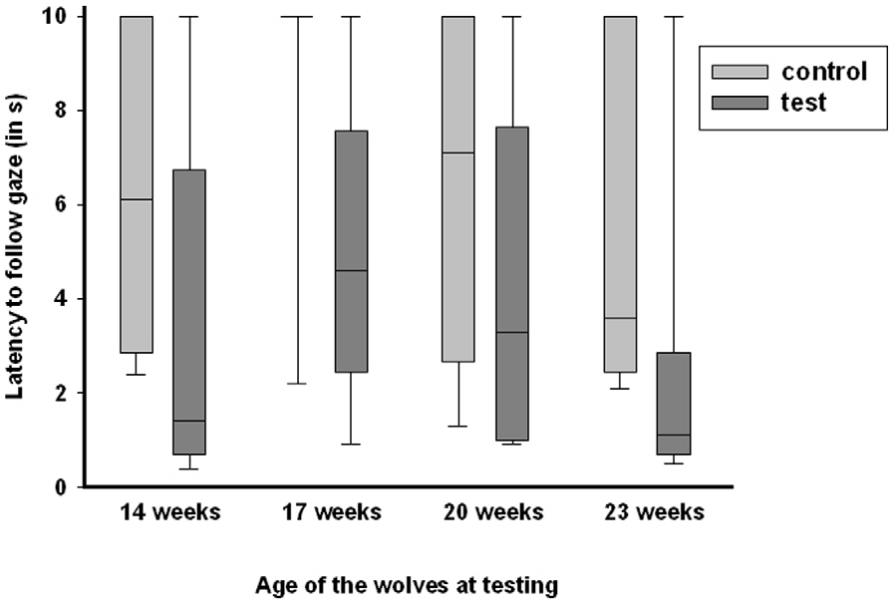
Box plots showing the latency of wolves to follow the gaze into distant space in seconds in the control and test trials at different ages of the animals. Shaded boxes represent the interquartile range, bars within shaded boxes are median values and whiskers indicate the 5th and 95th percentile.

We found that the wolves responded significantly more often in test than incontrol trials at 14 and 23 weeks of age within the first 2 seconds (McNemar test: week 14: N = 9, p<0.05; week 23: N = 9, p<0.05), but not at the age of 17 and 20 weeks (McNemar test: 17 weeks: N = 9, p>0.05, 20 weeks: N = 9, p>0.05) (Table 2). Moreover, when analyzing the latencies over the total cue presentation of 10 seconds, we found that at 14 and 17 weeks of age, the subjects had a significantly shorter latency to look into the demonstrated direction in the test trials than in the control trials (Wilcoxon matched-pairs signed ranks test: 14 weeks: N = 9 (2 ties), T = 0, p = 0.016, 17 weeks: N = 9 (2 ties), T = 1, p = 0.031, Figure 5). At 14 weeks, two animals showed no response neither in the test nor control trials; one of them still did not respond at 17 weeks of age. At 17 weeks of age, only one animal looked at in the direction of the demonstrated direction in the control trial. At 20 weeks of age, wolves only showed a trend towards a significant difference in response latency (Wilcoxon matched-pairs signed ranks test: N = 9, T = 6, p = 0.055, Figure 5). The last time the animals were tested at 23 weeks of age, all subjects but one followed the demonstrated gaze direction with a significantly shorter latency in the test trials than in the control trial (Wilcoxon matched-pairs signed ranks test: N = 9 (1 tie), T = 0, p = 0.008, Figure 5).

**Table 2:** Individual performance of the 9 wolves in the gaze following into distant space task in relation to both analyzed variables.

		14 weeks		17 weeks		20 weeks		23 weeks	
Ind	Sex	1/0	Lat	1/0	Lat	1/0	Lat	1/0	Lat
Ar	M	+	+	-	+	-	+	+	+
Ka	M	-	+	-	+	+	+	+	+
Sh	F	+	+	+	+	-	+	+	+
Ta	F	+	+	-	-	-	+	-	+
Na	M	+	+	-	+	+	+	-	+
Ge	M	+	+	+	+	-	-	+	+
Yu	F	-	-	-	-	-	+	+	+
Ap	M	+	+	-	+	-	+	+	+
Ch	M	-	-	-	-	-	-	+	+

1/0:+ =  following gaze in test but not control trial.

- =  following the gaze in both trials *or* in none ( = tie).

Lat:+ =  shorter latency in test than control.

- =  longer latency in test than control *or* no reaction in either trial (tie).

1/0 refers to whether or not the *first* detectable head turn of the subject followed the direction of the demonstrated head movement within the first two seconds of the cue presentation. Latency (Lat) refers to if the subject followed the gaze cue earlier in the test than the control trial.

#### 1b - Habituation

The latency to respond in the very first trial of the habituation test did not differ significantly from the latency to respond in the last gaze following test at the age of 23 weeks (Wilcoxon matched-pairs signed ranks test: N = 9 (1 tie), T = 16, p = 0.844) but did differ significantly from the responses in the control trials (Wilcoxon matched-pairs signed ranks test: N = 9 (1 tie), T = 0, p = 0.008).

Overall, when analyzing latencies, we found no significant decrease either in the median latency to respond between the first 5 trials and the second 5 trials (Wilcoxon matched-pairs signed ranks test: N = 9 (1 tie), T = 11, p = 0.383) (Figure 3b). Interestingly, also the three older animals (1.5 years of age) continued to respond across the 10 trials. Similar, no significant decline in the occurrence of response within the first 2 seconds after the gaze cue was given wasfound (Cochran's Q: Q_4_ = 14.06, N = 9, p>0.05).

### Discussion

The wolves followed the gaze of the human demonstrator into distant space significantly more often in the first 2 seconds and faster in the entire test condition compared to the control condition. This ability was already present at an age of 14 weeks when the wolves were tested the first time. In contrast to following an experimenter's gaze around a barrier, tracking the gaze direction of a human into distant space developed considerably earlier as has also been reported for ravens [21]. As argued in the introduction, it seems to confirm that simpler mechanisms underlie this form of gaze following in contrast to geometrical gaze follow.

Gaze following into distant space seems common in the animal kingdom and has been shown in five taxa of mammals, in three taxa of birds and a tortoise (see [32], for review). Moreover, gaze following seems to develop rather early during ontogeny with human infants showing a reliable response at approximately 10 months of age [2], [33], rhesus macaques (*Macaca mulatta)* at the age of 5.5 months, chimpanzees (*Pan troglodytes*) at the age of 3–4 years [25] but see [34] for a gaze following response at the age of 10 months] and ravens at the age of 8 to 15 weeks shortly after fledging [21]. Wolves showed a reliable response to the gaze following cue already at the age of 14 weeks. This is relatively early in comparison to the development of that ability in the other mammalian species studied so far and might have to do with the earlier independence of wolves compared to primates. At the age of 14 weeks, wolf pups already spend a lot of time out of the den and engage in complex interactions with their fellow pack members including playing where they should keep track of each other.

In contrast to our expectation, the wolves showed a lack of habituation in the gaze following task into distant space. One explanation for the missing habituation may be that we tested juvenile (n = 6) and adolescent (n = 3) wolves but not adults. Tomasello and colleagues [25] found that rhesus macaques and chimpanzees only habituate when fully adult. However, since our animals did habituate rapidly in the barrier task (see above), it is unlikely that age was the confounding factor. Another primate species, common marmosets, also failed to habituate to a series of 18 cues [10], indicating that there may be differences in habituation patterns and thus differences in openness for learning across species.

## General Discussion

Taken together, our results provide the first evidence that a non-primate mammalian species, the wolf, is also able to follow the gaze of others' not only into distant space but also around barriers. Moreover, while the wolves did not habituate to the gaze following cue into distant space within 10 trials, they rapidly habituated towards gaze following in the barrier task.

While the ontogeny of the two different gaze following abilities was in line with our expectation supporting the theoretical consideration that these two abilities may have different underlying cognitive mechanisms, habituation patterns differed from our expectations based on published data in corvids.

One possible explanation for these differences in habituation patterns across species may be that following gaze cues of other individuals has different functions for different taxa, suggesting that the ‘openness’ for learning is under different selection pressures [21]. For example, function might differ according to the type of cue being used in an experiment with look-up cues in birds and primates mainly being employed for predator detection, while look-aside cues in wolves are probably mainly important for distracting social and hunting information. While it may make sense to habituate to a look-up cue over short or long if no predator can be detected, habituation to a look-aside cue might not be very adaptive. Wolves live in closed social groups with social interactions changing constantly and rapidly. Thus, following the cue of another individual repeatedly might still be adaptive since it might reveal new information every time.

Overall, our data suggest that wolves are excellent at using conspecifics' as well as human gaze cues (if properly socialized) even to track gaze behind barriers, showing that this ability is not restricted to primate and corvid species. Relying on gaze cues to understand other individuals as intentional beings or, alternatively, to learn to use others' gaze cues, as predictors for their future behaviour, may be a crucial prerequisite for the highly cooperative social system in which wolves live. However, these new results raise questions as why dogs do not follow gaze into distant space [35] but do so in object choice tasks and call for further tests in dogs and wolves to get a better understanding of the effect of domestication on dogs' sensitivity towards human communicative cues [36]. Finally, the patterns of ontogeny and habituation found in our wolves provide further evidence that the underlying cognitive mechanisms in the two gaze following abilities differ.

## Supporting Information

Video S1 **Gaze following around the barrier.** The video illustrates a test and control trial of the gaze following around a barrier response of a wolf.
(M4V)

Video S2 **Gaze following into distant space.** The video illustrates a test and control trial of the gaze following response of a wolf.
(M4V)
